# Estimating the effects of body mass index and central obesity on stroke in diabetics and non‐diabetics using targeted maximum likelihood estimation: Atherosclerosis Risk in Communities study

**DOI:** 10.1002/osp4.447

**Published:** 2020-08-18

**Authors:** Hossein Mozafar Saadati, Yadollah Mehrabi, Siamak Sabour, Mohammad Ali Mansournia, Seyed Saeed Hashemi Nazari

**Affiliations:** ^1^ Department of Epidemiology, School of Public Health and safety Shahid Beheshti University of Medical Sciences Tehran Iran; ^2^ Department of Epidemiology and Biostatistics, School of Public Health Tehran University of Medical Sciences Tehran Iran; ^3^ Prevention of Cardiovascular Disease Research Center, Department of Epidemiology, School of Public Health and Safety Shahid Beheshti University of Medical Sciences Tehran Iran

**Keywords:** diabetes, obesity, stroke

## Abstract

**Objectives:**

The association of body mass index (BMI) with the risk of cardiovascular disease among diabetic patients is controversial. This study compared the effects of BMI and central obesity on stroke in diabetics and non‐diabetics using targeted maximum likelihood estimation.

**Materials and methods:**

This analysis included 12 725 adults aged 45–75 years, after excluding prevalence cases and participants with missing data, from the Atherosclerosis Risk in Communities study. Obesity was defined with BMI, waist circumference, waist‐to‐hip ratio (WHR), waist‐to‐height ratio (WHtR), body shape index (BSI) and body roundness index (BRI), which categorized all participants as obese and non‐obese. Generalized linear models and TMLE (with the tmle package) were used to estimate risk ratio (RR).

**Results:**

During 27 years of follow‐up, 1078 (8.47%) cases of stroke occurred. After adjustment for demographic, behavioural, biologic and central obesity variables, the effect of BMI decreased in both diabetics and non‐diabetics. The effect of BMI in diabetics was more attenuated, in full model, (RR: 1.04 [0.90, 1.20]) rather than non‐diabetics (RR: 1.11 [1.00, 1.24]). This attenuation was more related to biologic variables in non‐diabetics and central obesity in diabetics. With respect to central obesity, BSI (RR [95% CI]: 1.15 [0.96, 1.38]) and WHR (RR [95% CI]: 1.15 [0.87, 1.52]) had strongest and marginally significant effects for diabetics and BSI (RR [95% CI]: 1.10 [1.02, 1.20]) for non‐diabetics.

**Conclusions:**

Among diabetics, BSI and WHR indices were associated with a higher incidence of stroke. Future studies should consider how central obesity affects higher incidence of stroke among diabetics stratified by sex and age groups.

## 1 INTRODUCTION

Stroke is one of the important causes of mortality and disability worldwide.^1^, ^2^ Obesity is a recognized risk factor for many diseases, such as cardiovascular and cerebrovascular diseases.^2^ The association of obesity and stroke, however, is controversial. In this case, the body mass index (BMI) is the common index for obesity and overweight, which is unable to differentiate between excess fat mass and other body masses.^3^, ^4^, ^5^ For example, individuals with high BMI may have excess muscle mass instead of fat mass.^6^ Given the importance of visceral fat distribution for chronic diseases, waist circumference (WC), waist‐to‐hip ratio (WHR) and waist‐to‐height ratio (WHtR) have gained popularity for the measurement of obesity. These indices are closely related to central fat mass and can be used as indicators for central obesity.^7^

In addition, the contradictory results regarding the effect of obesity on stroke could be due to the lack of attention to the different fat distributions and obesity definitions in males and females.^8^, ^9^ Furthermore, previous studies have indicated a paradoxical effect of fat distribution in diabetics and non‐diabetics.^10^, ^11^, ^12^ Therefore, some studies have shown an inverse association between BMI and stroke, and some studies have shown that the central obesity is a useful predictor of stroke in diabetics.

Model misspecification is another problem for the assessment of this complex and multifactorial relationship, especially when observational studies are used for causal inference.^13^, ^14^ Targeted maximum likelihood estimation (TMLE) is a two‐stage estimator that reduces the bias for the estimation of the target parameters if either exposure or outcome models are estimated consistently.^14^, ^15^ Furthermore, this estimator is based on causal assumptions under which observational data may emulate inference from a perfect randomized trial, allowing us to evaluate the nearest causal and true effects.^16^ Moreover, TMLE is known as a double‐robust method and naturally integrates loss‐based super learning, which increases the chance to reduce bias due to model misspecification.^16^

The current study was designed to examine the unbiased association of BMI and central obesity with the risk of stroke separately for diabetics and non‐diabetics in the Atherosclerosis Risk in Communities (ARIC) cohort study using the TMLE method. It was hypothesized that diabetic participants with central obesity are more at risk of developing a stroke.

## 2 MATERIALS AND METHODS

### 2.1 Study design and participants

The ARIC study is a prospective cohort study designed to evaluate the risk factors of atherosclerotic disease. ARIC is a community‐based cohort comprising participants from four U.S. communities (Washington County, Maryland; Jackson, Mississippi; Forsyth County, North Carolina; and the suburbs of Minneapolis, Minnesota). In 1987–1989 (Visit 1), 15 792 males and females aged 45–64 were recruited and completed the baseline clinic examination (Visit 1). Then, every 3 years, all participants were invited to a follow‐up examination during 1990–1992, 1993–1995 and 1996–1998. Cohort participants were selected by probability sampling. In these communities, all age‐eligible persons were selected as potential cohort participants. The baseline examination evaluated cardiovascular conditions and assessed the related risk factors. After each visit, a telephone questionnaire was administered annually, and medical conditions were identified by the annual questionnaire. Response rates for the successive examinations were 93%, 86% and 80%, respectively. The institutional review boards approved the ARIC study protocol by each participating study field centre, and informed consent was obtained from participants at each study visit. Details of this study are described elsewhere.^17^ In the current study, the baseline data, the exposure and covariates values in Visit 1, and all outcome to 2014, were included. The definition of type 2 diabetes occurrences was based on blood glucose level ≥200 mg dl^−1^ or blood glucose level after 8 h or more of fasting time ≥140 mg dl^−1^. According to this cut point, all participants were divided into diabetic and non‐diabetic groups.

### 2.2 Measurements

#### 2.2.1 Exposure

Six anthropometric indices to define obesity and (standardized) central obesity were characterized. The anthropometric measures including height and the circumference of the waist and hip to the nearest centimetre on fasting participants wearing hospital standard scrub suit were measured in ARIC study. Obesity was defined as BMI (weight [kg]/height [m^2^]) ≥ 30 kg m^2^. Central obesity was defined as WC ≥ 102 cm in males and ≥88 cm in females, WHR ≥ 0.9 in males and ≥0.85 in females and WHtR ≥ 0.5. Standardized central obesity was defined as body shape index (BSI) (WC/[BMI^2/3^ × height^1/2^]) ≥ 0.08 and body roundness index (BRI) (364.2 − (365.5 * square root(1 − ((WC/2π)^2)/(0.5 * Height)^2)))) ≥ 4.^18^, ^19^ Because there is no universal agreement regarding the BSI and BRI cut point, it was also evaluated based on best threshold cut‐off value in receiver operating characteristic (ROC) curve.

#### 2.2.2 Outcome and covariates

A definite or probable stroke that occurred by 31 December 2014 (after 27 years of follow‐up) was considered as outcome (binary outcome [0, 1]) in the present study. Data were collected by annual telephone interviews that listed all hospitalizations during the past year. In addition, all local hospitals provided lists of stroke occurrences. This outcome was identified by the presence of related hospital discharge codes (ICD‐9 codes 430–438 until 1997 and ICD‐9 codes 430–436), presence of stroke findings on a computerized tomography (CT) or magnetic resonance imaging (MRI) report or by death certificates.^20^ All included covariates were categorized in three demographic, behavioural and biologic dimensions as potential confounders. These covariates included age, sex, race, education level, resident centre, cigarette smoking status, drinker status, total physical activity score, total calorie intake (kcal), hypertension, plasma lipids (mg dl^−1^) and the history of stroke at the baseline. The plasma lipids included cholesterol, high‐density lipoprotein (HDL) cholesterol and triglyceride. In addition, waist and hip circumference were included in full models for evaluating the possible mediation effect, as appropriate.

#### 2.2.3 Causal diagram and notations

**FIGURE 1:**
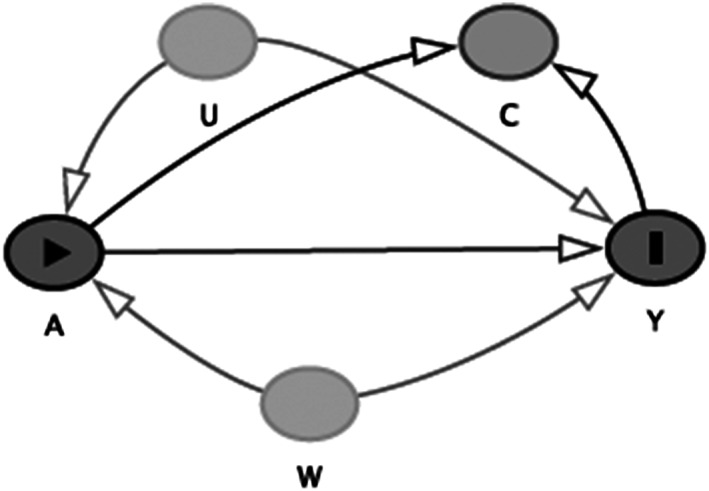
Causal diagram for the effect of obesity on Stroke. A, exposure (obesity); Y, outcome (Stroke); W, measured confounder; C, missing outcome; U, unmeasured confounder

A directed acyclic graph (DAG) in Figure 1 represents the associations between the exposure and outcome and other covariates. In this method, the data are represented in data structure given by *O* = (*W*, *A*, *Δ*, *ΔY*_*A*_), where *W* is a vector of measured baseline covariates, *A* is an exposure variable, delta is a missing mechanism for outcome of interest in which *Δ* = 1 indicates the outcome is observed, and *Δ* = 0 indicates the outcome is missing, and *Y* is the study outcome. In this study, *W* is all listed potential confounders, *A* is obesity based on different indices, *Δ* is the missing mechanism of outcomes that is explained in statistical analysis section, and *Y* is the occurrence of stroke.

### 2.3 Statistical analysis

Descriptive statistics were used to describe the participants (mean ± SD for continuous variables and number and percent for categorical variables). An independent *t*‐test analysis was used to examine the statistical differences in continuous covariates between two levels of stroke. In addition, the *χ* ^2^ test was used to examine the associations of categorical variables with stroke. *P* < .05 was considered statistically significant.

The TMLE was used to quantify the relationship between BMI and central obesity and stroke. TMLE, as a double‐robust estimator, which uses both outcome and exposure models, was implemented in several steps. The statistical and mathematical details of this method and related components are described elsewhere.^15^, ^16^

The super learner approach is an ensemble machine learning approach that uses cross‐validation to select an optimal statistical model from among many candidate models. In this study, a super learner algorithm with three algorithms, including generalized linear model (GLM), stepwise GLM and interaction GLM, was used. Model misspecification may be present in many situations. In these situations, the models do not account for everything, and this may result in biased estimations. Thus, recently, researchers have developed robust methods to reduce this problem. Double‐robust methods have the advantage of using both exposure and outcome models that remain consistent if either exposure or outcome models are estimated consistently. In addition, TMLE can use the super learner machine learning algorithms that result in protecting against bias.

The exposure variable (obesity) was characterized dichotomously; values above the defined cut‐off were considered as ‘obese’ and the other ones as ‘non‐obese.’ The missing mechanism (outcome missing at follow‐up) was defined as the occurrence of a competing event (total mortality of all other causes) or loss to follow‐up before the occurrence of the stroke.^21^

To assess the possible mediation effect of biological and central obesity covariates in the association between obesity and stroke, this relationship was evaluated in four models in diabetics and non‐diabetics for six obesity indices. In these four models, the effect of BMI was assessed adjusting for demographic, behavioural and biological covariates and central obesity indices (waist and hip circumference), respectively. For central obesity indices, the fourth model was only adjusted for Model 3 and hip circumference. To evaluate the confounding effects of behavioural covariates, Models 2 and 1 were compared. To evaluate the possible mediation effect through biological variables in this pathway, Models 3 and 2 were compared. Comparison of Models 4 and 2 shows how much of the BMI effect is possibly explained by central obesity indices (waist and hip circumference) and biological variables, and comparison of Models 4 and 3 shows how much of the BMI effect is explained through the possible mediation effect of central obesity indices. For central obesity indices, comparison of Models 4 and 3 shows how much of the effects is explained through the possible mediation effect of hip circumference.

The sensitivity analyses were used in three ways. First, because complete data were not available for all the covariates, the sensitivity analyses were conducted to compare the complete case models and missing imputation models. Missing covariate data were completed by multiple imputation with the Amelia package in R, and the adaptive methods for rounding the binary variables, introduced by Bernaards et al., were used, in spite of the default rounding of Amelia.^22^ Simulation results show the superiority of adaptive rounding in an unpublished study by the authors. The Rubin's rules were used to calculate point estimates and standard errors in five imputed datasets.^23^

Second, the sensitivity analyses were conducted to compare the full model and the reduced model without the following biological variables: hypertension, diabetes mellitus, plasma lipids (mg dl^−1^) and/or central obesity indices, in order to investigate the mediation effects of biological covariates.

And third, the sensitivity analyses were conducted to compare TMLE with super learner algorithms (as the TMLE method) and GLMs (as the conventional method). The GLMs and TMLE models were fit and included all listed covariates as confounders and obesity (with six definitions) as binary exposure. The performance of the models was then compared based on the amount of bias (mean absolute error) and mean root square error.

Finally, the risk ratios and the corresponding confidence intervals were calculated. The variance was estimated using the influence‐curve method. Internal validation was performed in the super learner model by cross‐validation. The analysis was done using the multivariate logistic regression for GLM models and the tmle package for TMLE in R version 3.6.1.

For accessing the data, we signed an RMDA (NHLBI Research Materials Distribution Agreement), which is available upon request. Approval of the study by the Institutional Review Board of Shahid Beheshti University of Medical Sciences (IR. SBMU.PHNS.1396.152) was obtained on 14 March 2018.

## 3 RESULTS

### 3.1 Participant characteristics

Baseline characteristics of the study participants are shown in Table 1. Of the 14 983 participants at baseline, 12 725 participants met the inclusion criteria. All participants with a history of any stroke disorder (prevalence cases) and those with missing data at baseline were excluded. Regarding the BMI, compared with participants without obesity, participants with obesity were more likely to be female, Black, have lower education and annual family income and were less likely to have health insurance. In addition, this group had higher prevalence of hypertension, antihypertensive medication use, diabetes mellitus and lower HDL cholesterol. With respect to central obesity indices, participants with obesity were more likely to be male, White, have lower education and annual family income and were less likely to have health insurance compared with participants without obesity. During a median 27 years of follow‐up, 1078 (8.47%) males and females experienced stroke. The cumulative incidence rate of stroke was 84.72 per 1000 (95% CI: 79.77, 89.89).

**TABLE 1:** Baseline characteristics by stroke occurrence of participant in the ARIC study, 1987–2014

Characteristic		Stroke occurrence		*P* value^a^
		Yes	No	
Categorical variables		No. %	No. %	
Sex	Female	555 (51.48)	6375 (54.74)	.040
	Male	523 (48.52)	5272 (45.26)	
Race	White	709 (65.77)	9137 (78.45)	<.001
	Black	369 (34.23)	2510 (21.55)	
Education	Basic	348 (32.28)	2457 (21.10)	<.001
	Intermediate	427 (39.61)	4865 (41.77)	
	Advanced	303 (28.11)	4325 (37.13)	
Family income (per year)	Less than $16 000	338 (31.35)	2357 (2.24)	<.001
	$16 000–$50 000	574 (53.25)	6147 (52.78)	
	More than $50 000	166 (15.40)	3143 (26.99)	
Drinker status	Current drinker	538 (49.91)	6800 (58.38)	<.001
	Former drinker	236 (21.89)	2126 (18.25)	
	Never drinker	304 (28.20)	2721 (23.36)	
Cigarette smoking status	Current smoker	330 (30.61)	2947 (25.30)	.001
	Former smoker	332 (30.80)	3868 (33.21)	
	Never smoker	416 (38.59)	4832 (41.49)	
Health insurance	No	144 (13.36)	957 (8.22)	<.001
	Yes	934 (86.64)	10 690 (91.78)	
Family history of CVD	No	418 (38.78)	4986 (42.81)	.010
	Yes	660 (61.22)	6661 (57.19)	
Hypertension	No	605 (56.12)	8547 (73.38)	<.001
	Yes	473 (43.88)	3100 (26.62)	
Antihypertensive medicine	No	625 (57.98)	8338 (71.59)	<.001
	Yes	453 (42.02)	3309 (28.41)	
Diabetes mellitus	No	876 (81.26)	10 650 (91.44)	<.001
	Yes	202 (18.74)	997 (8.56)	
BMI	Non‐obese	721 (66.88)	8659 (74.35)	<.001
	Obese	357 (33.12)	2988 (25.65)	
WC	Non‐obese	436 (40.45)	5596 (48.05)	<.001
	Obese	642 (59.55)	6051 (51.95)	
WHR	Non‐obese	169 (15.68)	2597 (22.30)	<.001
	Obese	909 (84.32)	9050 (77.70)	
WHtR	Non‐obese	126 (11.69)	1899 (16.30)	<.001
	Obese	952 (88.31)	9748 (83.70)	
BSI	Non‐obese	339 (31.45)	4469 (38.37)	<.001
	Obese	739 (68.55)	7178 (61.63)	
BRI	Non‐obese	261 (24.21)	3756 (32.25)	<.001
	Obese	817 (75.79)	7891 (67.75)	
Continues variables		Mean (SD)	Mean (SD)	
Age, years		55.94 ± 5.53	54.03 ± 5.74	<.001
Physical activity (work)		2.12 ± 0.97	2.19 ± 0.93	.036
Physical activity (sport)		2.36 ± 0.75	2.46 ± 0.79	<.001
Physical activity (leisure time)		2.30 ± 0.58	2.38 ± 0.56	<.001
Total energy intake (kcal)		1643.5 ± 626.7	1617.8 ± 603.3	.183
Saturated fatty acid (%kcal)		12.11 ± 3.04	12.01 ± 2.98	.328
Total cholesterol mg dl^−1^		5.69 ± 1.14	5.54 ± 1.07	<.001
Triglyceride mg dl^−1^		1.62 ± 1.00	1.48 ± 1.01	<.001
HDL cholesterol mg dl^−1^		1.27 ± 0.42	1.33 ± 0.44	<.001

Abbreviations: ARIC, Atherosclerosis Risk in Communities; BMI, body mass index; BRI, body roundness index; BSI, body shape index; CVD, cardiovascular disease; HDL, High‐density lipoprotein cholesterol; kcal, kilocalorie; LDL, low‐density lipoprotein cholesterol; mg dl^−1^, milligrams per deciliter; NO. %, number and percentage of participants in each group of stroke; SD, standard deviation; WC, waist circumference; WHR, waist‐to‐hip ratio; WHtR, waist‐to‐height ratio; %kcal, percentage of kilocalorie.

^a^ *P* value was based on *χ* ^2^ test and independent *t* test for categorical and continues variables, respectively.

The performance of the models based on bias and mean root square error showed better results for super leaner models. Hence, all results are provided based on the super leaner model.

### 3.2 Outcome evaluation by diabetic groups (diabetics and non‐diabetics)

**FIGURE 2:**
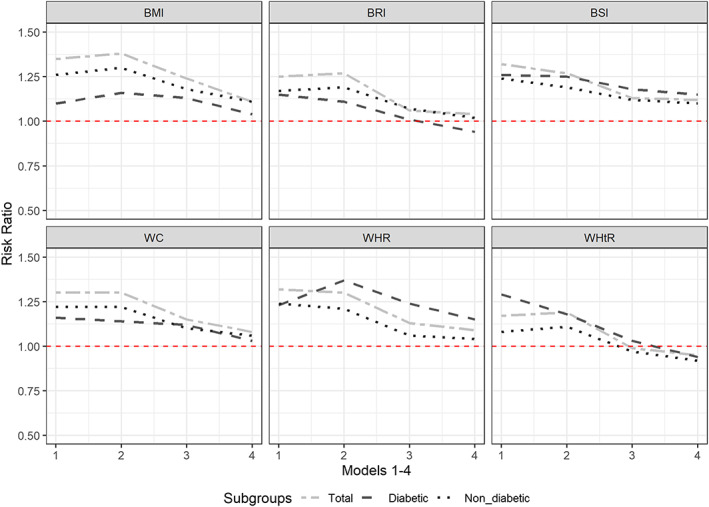
Decreasing risk of stroke for six obesity indices after adjustment for demographic, behavioural, biologic and central obesity variables in four models, respectively, in communities (ARIC) study (1987–2014), in total, diabetics, non‐diabetics, males and females. BMI, body mass index; WC, waist circumference; WHR, waist‐to‐hip ratio; WHtR, waist‐to‐height ratio; BSI, body shape index; BRI, body roundness index

The risk ratios with 95% confidence intervals for six obesity indices in four adjusted models are presented in Tables 2, 3, 4 and Figure 2 for all participants, diabetics and non‐diabetics, respectively. The obtained effect for BMI adjusted for demographic covariates was 1.10 in diabetics, which increased to 1.16 after adjusting for behavioural covariates (negative confounding role of behavioural variables). This effect was attenuated by 12% (decreased toward the null) after including the waist and hip circumference and biological variables in the fourth model. In non‐diabetics, this effect was attenuated by 19% after including the waist and hip circumference and biological variables.

**TABLE 2:** Estimated risk ratios of stroke by baseline body mass index and central obesity of total participants (*N* = 12 725) in the ARIC study, 1987–2014, based on targeted maximum likelihood estimation with super learner modelling

Index	Model 1	Model 2	Model 3	Model 4
	RR (95% CI)	RR (95% CI)	RR (95% CI)	RR (95% CI)
BMI	1.35 (1.23, 1.47)	1.38 (1.27, 1.50)	1.24 (1.14, 1.35)	1.11 (1.02, 1.21)
WC^a^	1.30 (1.20, 1.41)	1.30 (1.20, 1.40)	1.15 (1.07, 1.24)	1.08 (1.01, 1.17)
WHR^a^	1.32 (1.20, 1.44)	1.30 (1.19, 1.41)	1.13 (1.04, 1.22)	1.09 (1.01, 1.18)
WHtR^a^	1.17 (1.06, 1.30)	1.19 (1.08, 1.31)	0.99 (0.92, 1.07)	0.95 (0.88, 1.02)
BSI^a^	1.32 (1.22, 1.43)	1.27 (1.18, 1.37)	1.13 (1.06, 1.22)	1.12 (1.04, 1.20)
BRI^a^	1.25 (1.15, 1.36)	1.27 (1.17, 1.37)	1.06 (0.99, 1.14)	1.04 (0.96, 1.11)

*Note*: Model 1: adjusted for demographic variables and family history; Model 2: Model 1 plus behavioural variables; Model 3: Model 2 plus biologic variables; Model 4: Model 3 plus waist circumference and hip circumference.

Abbreviations: ARIC, Atherosclerosis Risk in Communities; BMI, body mass index; BRI, body roundness index; BSI, body shape index; CI, confidence interval; RR, risk ratio; WC, waist circumference; WHR, waist‐to‐hip ratio; WHtR, waist‐to‐height ratio.

^a^ Adjusted for Model 3 plus hip circumference.

**TABLE 3:** Estimated risk ratios of stroke occurrence by baseline obesity of males and females in the ARIC study, 1987–2014 (Model 4)

Index	Model 4	
	Males	Females
	RR (95% CI)	RR (95% CI)
BMI	1.06 (0.92, 1.22)	1.08 (0.94, 1.24)
WC^a^	1.22 (1.09, 1.36)	1.02 (0.92, 1.12)
WHR^a^	1.14 (0.97, 1.33)	1.07 (0.96, 1.19)
WHtR^a^	0.89 (0.78, 1.02)	0.98 (0.88, 1.09)
BSI^a^	1.17 (1.04, 1.30)	1.15 (1.03, 1.28)
BRI^a^	0.98 (0.88, 1.09)	1.15 (1.03, 1.27)

*Note*: Model 4: adjusted for demographic variables, family history, behavioural variables and biologic variables plus waist circumference and hip circumference.

Abbreviations: ARIC, Atherosclerosis Risk in Communities; BMI, body mass index; BRI, body roundness index; BSI, body shape index; CI, confidence interval; RR, risk ratio; WC, waist circumference; WHR, waist‐to‐hip ratio; WHtR, waist‐to‐height ratio.

^a^ Adjusted for hip circumference.

**TABLE 4:** Estimated risk ratios of stroke by baseline body mass index and central obesity, for diabetics (*N* = 1199) and non‐diabetics (*N* = 11 526) in the ARIC study, 1987–2014, based on targeted maximum likelihood estimation with super learner modelling

Index	Subgroups	Model 1	Model 2	Model 3	Model 4
		RR (95% CI)	RR (95% CI)	RR (95% CI)	RR (95% CI)
BMI	Diabetic	1.10 (0.94, 1.28)	1.16 (1.00, 1.36)	1.13 (0.97, 1.31)	1.04 (0.90, 1.20)
	Non‐diabetic	1.26 (1.13, 1.41)	1.30 (1.18, 1.44)	1.18 (1.06, 1.31)	1.11 (1.00, 1.24)
WC^a^	Diabetic	1.16 (0.96, 1.40)	1.14 (0.94, 1.40)	1.12 (0.93, 1.35)	1.03 (0.86, 1.24)
	Non‐diabetic	1.22 (1.11, 1.34)	1.22 (1.12, 1.33)	1.10 (1.01, 1.20)	1.06 (0.97, 1.15)
WHR^a^	Diabetic	1.23 (0.91, 1.66)	1.37 (0.99, 1.90)	1.24 (0.92, 1.69)	1.15 (0.87, 1.52)
	Non‐diabetic	1.24 (1.11, 1.37)	1.21 (1.10, 1.32)	1.06 (0.97, 1.15)	1.04 (0.96, 1.14)
WHtR^a^	Diabetic	1.29 (1.00, 1.65)	1.18 (0.94, 1.48)	1.03 (0.83, 1.26)	0.94 (0.76, 1.16)
	Non‐diabetic	1.08 (0.97, 1.20)	1.11 (1.00, 1.23)	0.97 (0.88, 1.07)	0.92 (0.84, 1.01)
BSI^a^	Diabetic	1.26 (1.03, 1.54)	1.25 (1.02, 1.53)	1.18 (0.98, 1.41)	1.15 (0.96, 1.38)
	Non‐diabetic	1.24 (1.13, 1.36)	1.19 (1.09, 1.29)	1.12 (1.03, 1.21)	1.10 (1.02, 1.20)
BRI^a^	Diabetic	1.15 (0.89, 1.48)	1.11 (0.87, 1.42)	1.01 (0.79, 1.30)	0.94 (0.74, 1.19)
	Non‐diabetic	1.17 (1.06, 1.29)	1.19 (1.09, 1.30)	1.07 (0.98, 1.16)	1.02 (0.94, 1.11)

*Note*: Model 1: adjusted for demographic variables and family history; Model 2: Model 1 plus behavioural variables; Model 3: Model 2 plus biologic variables; Model 4: Model 3 plus waist circumference and hip circumference.

Abbreviations: ARIC, Atherosclerosis Risk in Communities; BMI, body mass index; BRI, body roundness index; BSI, body shape index; CI, confidence interval; RR, risk ratio; WC, waist circumference; WHR, waist‐to‐hip ratio; WHtR, waist‐to‐height ratio.

^a^ Adjusted for Model 3 plus hip circumference.

Regarding the central obesity, the obtained effect for WC index adjusted for demographic covariates was 1.16 in diabetics, which decreased to 1.14 after adjusting for behavioural covariates (positive confounding role of behavioural variables). This effect was attenuated by 11% after including the hip circumference and biological variables. With respect to WHR index, the obtained effect adjusted for demographic covariates was 1.23 in diabetics, which increased to 1.37 after adjusting for behavioural covariates. This effect was attenuated by 22% after including the hip circumference and biological variables. For WHtR index, the estimated effect for Model 1 was 1.29, which decreased to 1.18 after adjusting for behavioural covariates (positive confounding role). This effect was reversed to 0.94 after including the waist and hip circumference and biological variables in the fourth model.

The effect of BSI index decreased for both diabetics and non‐diabetics after fitting the fourth model; however, there was still a harmful effect in both groups. Regarding the BRI index, the effect in diabetics and non‐diabetics was 1.15 and 1.17, which decreased toward the null, 0.94 and 1.02, respectively. Finally, the strongest index for diabetics in the full model was WHR (RR [95% CI]: 1.15 [0.87, 1.52]) and BSI (RR [95% CI]: 1.15 [0.96, 1.38]), and for non‐diabetics, it was BMI (RR [95% CI]: 1.11 [1.00, 1.24]) and BSI (RR [95% CI]: 1.10 [1.02, 1.20]) (Table 4).

### 3.3 Outcome evaluation for all participants and by sex (males and females)

**FIGURE 3:**
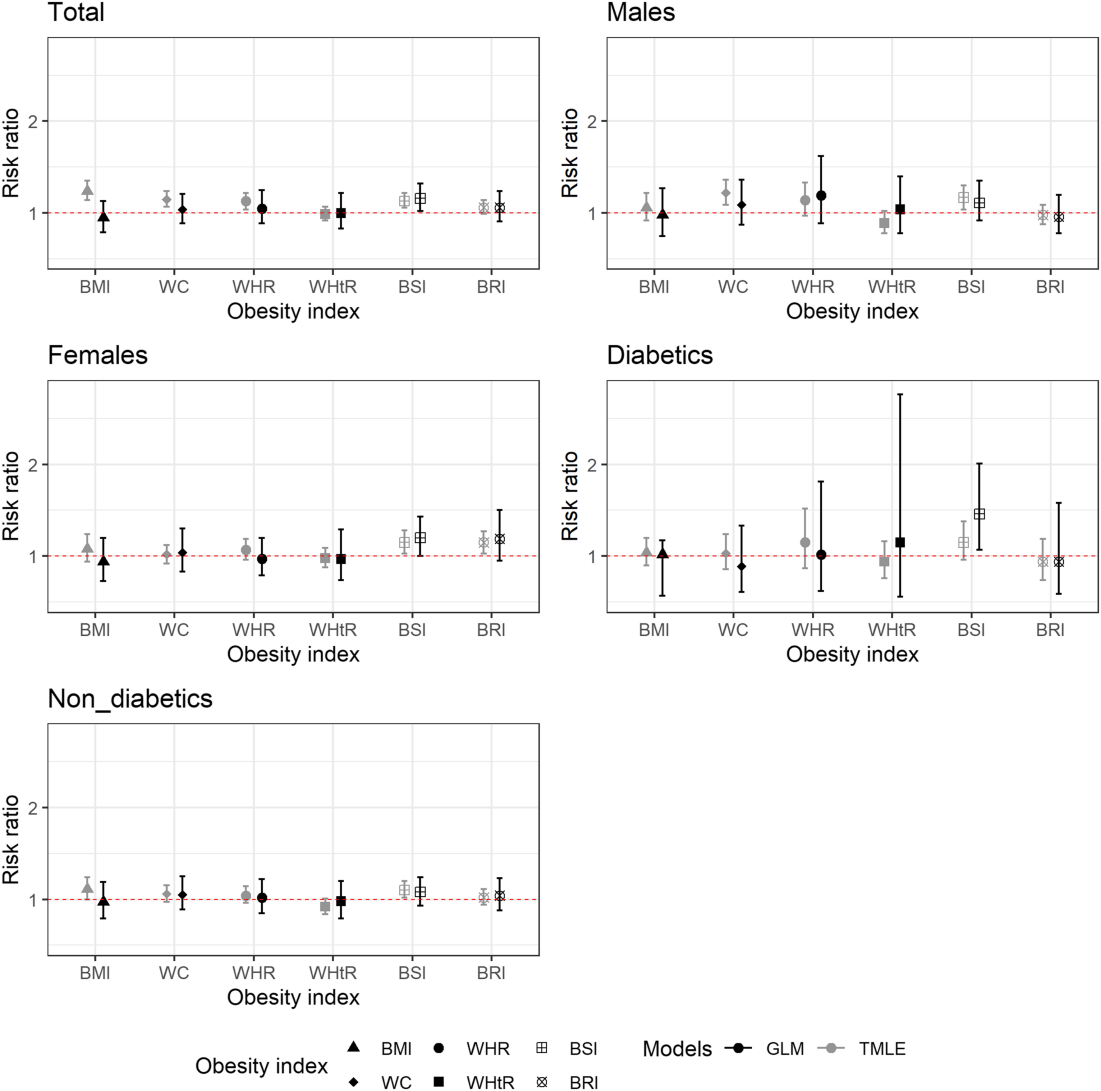
Comparison of risk ratios of stroke for six obesity indices based on targeted maximum likelihood estimation with super learner modelling (tmle) and generalized linear model (glm) in the atherosclerosis risk in communities (ARIC) study (1987–2014). BMI, body mass index; WC, waist circumference; WHR, waist‐to‐hip ratio; WHtR, waist‐to‐height ratio; BSI, body shape index; BRI, body roundness index

The risk ratios with 95% confidence intervals estimated by TMLE for six obesity indices adjusted for all listed covariates are presented in Figure 3 and Table 2 for all participants and in Table 3 for males and females. The results (the fourth model as mentioned in the method [adjusted for demographic, behavioural and biological covariates and central obesity indices as appropriate]) for all participants show that the more harmful and significant effects were related to BSI (RR [95% CI]: 1.12 [1.04, 1.20]), BMI (RR [95% CI]: 1.11 [1.02, 1.21]), WHR (RR [95% CI]: 1.09 [1.01, 1.18]) and WC (RR [95% CI]: 1.08 [1.01, 1.17]), respectively. Besides that, the effects for other indices, except for WHtR, were marginally significant. WHtR had a protective effect but not significant. The findings for males and females provided somewhat different results. For males, the effects of WC (RR [95% CI]: 1.22 [1.09, 1.36]), BSI (RR [95% CI]: 1.17 [1.04, 1.30]) and WHR (RR [95% CI]: 1.14 [0.97, 1.33]) were more harmful and significant. For females, the effects of BSI (RR [95% CI]: 1.15 [1.03, 1.28]) and BRI (RR [95% CI]: 1.15 [1.03, 1.27]) indices were more harmful and significant.

### 3.4 Sensitivity analyses

In sensitivity analyses, the results of complete data and imputed data had an ignorable difference. The second sensitivity analysis comparing models with and without biological variables exhibited that the effects were sensitive to exclusion of these covariates. These results can indicate the mediation effects of biological factors (Tables 2 and 4). The third sensitivity analysis showed that the results of the TMLE method were more precise than the GLM method. The point estimates of these two methods were different, and all the confidence intervals for the TMLE method were more precise (Figure 3).

## 4 DISCUSSION

In this large cohort study, the TMLE method was used to estimate the association of BMI and central obesity with stroke in diabetics and non‐diabetics. Furthermore, this association for all participants and sex groups was evaluated. It is worth mentioning that model misspecification and biased estimation are two important problems in conventional statistical methods, especially for observational data. To solve these problems, this study used the TMLE method that considers these limitations and makes causal assumptions that allow us to evaluate the nearest causal and true effects. Generally, regarding central obesity, the WHR and BSI indices have the strongest and marginally significant effects of stroke occurrence in diabetics, and the BSI does so for non‐diabetics. Furthermore, the effects of BMI were attenuated to the null after adjusting for central obesity in both diabetics and non‐diabetics. With respect to the effects in males, females and all participants, the results of the TMLE method (the fourth model) were more precise than those based on conventional models and showed that the strongest effect was related to BSI and BMI for all participants: WC, BSI and WHR for males and BSI and BRI for females.

The results of the current study agree with those of a Mendelian randomization study on the effect of BMI on ischaemic stroke, for all participants.^24^ The findings of that study confirmed a weak and marginally significant result. However, this relationship was not assessed separately among males and females or diabetics and non‐diabetics. In addition, only BMI was assessed as an obesity index, which is subject to some limitation.

Consistent with our study, regarding the association between BMI and stroke in diabetics, previous studies have found a null or inverse association between BMI and total stroke.^11^, ^25^ However, some studies have shown inconsistent results.^11^, ^26^ On the other hand, regarding the association between central obesity and stroke in diabetics, previous studies have found that central obesity in diabetics plays an important role in causal pathway of stroke.^27^, ^28^ These contradictions seem to arise largely because of misclassification of persons with and without obesity by BMI and misspecification of the models. The misspecification problem could be minimized by the TMLE method, which uses super learning algorithms.^16^ The limitations of BMI were inherently related to the formula and measurement methods. Previous studies confirm that the causal pathways from obesity to different vascular diseases are very complex, and one obesity index cannot explain all of these pathways.^6^, ^29^ BMI cannot accurately discriminate the distribution of body masses that seems to have a causal relation with many vascular diseases. Other obesity indices that have more ability to differentiate the fat, muscle and bone mass can better explain these causal pathways.^6^, ^7^, ^18^, ^19^ Furthermore, the fat distribution and body shape are different in males and females, and several studies have confirmed the necessity of separate estimations of this relationship for males and females.^30^ Besides, the researcher should consider the mediation role of the biological risk factors confirmed in previous studies, separately for males and females.^31^, ^32^

It is worth noting that several studies in this field have been conducted using ARIC data; however, many of them have had some limitations that lead to biased and contradictory results.^33^ These studies are limited by the analysis methods, the covariates included in the models and misspecification of the models that is common.

Before interpreting and concluding the results, the strengths and limitations of this study need to be addressed. In this study, the double‐robust method was used, which consistently estimates the parameters under a semiparametric model when one of two (exposure and outcome) models is correctly specified, regardless of which, while most previously published studies used conventional methods for their analyses, which may be associated with estimation error and model misspecification.^11^, ^26^ In addition, different indices of obesity were used to determine the best index that defines the obesity concept for all participants and for each sex group. Furthermore, the missing mechanism of outcome was taken into account for better estimation of true effects. In contrast, this package only takes into account one phase of exposure and covariates estimation and is unable to consider the time‐varying confounders. The authors aim to consider this issue in future studies. In addition, in future studies, these associations should be evaluated in diabetics and non‐diabetics, separately for males and females.

In summary, the findings of our study indicated that the effect of different fat distribution was different between diabetics and non‐diabetics. Moreover, this inconsistency was shown for males and females, and previous studies have shown that females commonly have a higher percentage of body fat than males.^9^, ^30^ Previous findings were confirmed by considering the results of BMI and central obesity indices. WC and WHR, which are more related to waist girth, provide a stronger and more significant effect for males and BSI and BRI that consider other body measures and fatty mass do so for females.

## 5 CONCLUSION

From the present study, it can be generally concluded that among the measures of obesity, central indices had better prediction and stronger association with the incidence of stroke in diabetics. In sex groups, the WC and WHR indices in males and the BSI and BRI indices in females were more appropriate indices for definition of obesity as a risk factor for stroke.

## CONFLICT OF INTEREST

The authors declare no conflict of interest.
